# Unraveling the mechanism of mulberry leaf in alleviating hyperuricemia: key role of kaempferol by modulating AKT pathway and gut-kidney axis

**DOI:** 10.3389/fmicb.2026.1752775

**Published:** 2026-01-22

**Authors:** Jiawei Huang, Qianqian Wang, Xiaowen Guo, Yuanyuan Niu, Junhong Huang, Boyi Zhang, Zixuan Guo, Zilong Wang, Shuying Feng

**Affiliations:** 1 Medical College, Henan University of Chinese Medicine, Zhengzhou, China; 2 Henan Engineering Research Center for Chinese Medicine Foods for Special Medical Purpose, Zhengzhou, China

**Keywords:** 16S rRNAsequencing, Akt signaling pathway, hyperuricemia, *in vivo* study, kaempferol, moleculardynamics simulations, network pharmacology

## Abstract

**Background:**

Mulberry leaf (*Morus alba L*.) is an edible plant that has been found to have medicinal effects in the treatment of hyperuricemia (HUA). The bioactive compounds of mulberry leaf and their mechanisms of action have not been determined yet.

**Methods:**

*In-silico* methodologies were used to identify bioactive compounds and to determine the underlying mechanisms of mulberry leaf. In order to verify the biochemical mechanism and intestinal microbiota, *in vivo* experiments were conducted.

**Results:**

Kaempferol was identified as the principal bioactive compound, while the key targets were AKT1 and TNF. Molecular docking and dynamics simulations revealed that AKT1-kaempferol and TNF-kaempferol complexes showed strong and stable binding pattern after a 100 ns simulation. *In vivo* studies demonstrated that kaempferol exerted significant anti-HUA effects. Specifically, kaempferol reduces AKT expression and phosphorylation, which may in turn reduces the oxidative stress and inflammatory pathways and signal transmission of the kidneys. Meanwhile, the application of kaempferol attenuated gut microbiota dysbiosis caused by HUA.

**Conclusion:**

Kaempferol may regulate UA metabolism and inflammatory injury by modulating the AKT signaling pathway, and exert its effects on the gut-kidney axis and restoring gut microbiota composition.

## 1 Introduction

Hyperuricemia (HUA) is a metabolic disorder primarily characterized by elevated serum uric acid (UA) levels, which is often accompanied by hyperreactive oxidative stress and inflammatory responses (Yang et al., 2019b; Wang et al., 2022). Current pharmacological treatments for HUA include xanthine oxidase inhibitors such as allopurinol and febuxostat, and uricosuric agents like benzbromarone. Despite their efficacy in reducing UA levels, the long-term use of these agents is often associated with significant adverse effects, including hepatotoxicity, nephrotoxicity, cardiovascular complications, and severe hypersensitivity reactions (Yokose et al., 2019), which substantially limit their clinical applicability. Therefore, the development of safer and longer-acting therapeutic strategies for HUA is urgently needed.

Traditional Chinese medicines (TCM) have a long-standing history of clinical use and have demonstrated beneficial effects in the treatment of various metabolic disorders, such as hypertension, diabetes, and atherosclerosis (Huang et al., 2022; Yang et al., 2022; Zhang et al., 2022a). Mulberry leaf (*Morus alba L*.), a commonly used TCM food source in China, is consumed in various forms including tea infusions, stir-frying and deep-frying (Makchuay et al., 2023). Its bioactive compounds mainly include flavonoids, phenolic acids, coumarins, and polysaccharides (Shi et al., 2022). Recent studies have highlighted the potential of mulberry leaf in alleviating HUA. The ethyl acetate extract of mulberry leaf has been shown to regulate both UA production and excretion, leading to a significant reduction in serum UA levels (Yao et al., 2019; Wang et al., 2025). Additionally, the polysaccharide fractions exhibit strong antioxidant activity by scavenging hydroxyl radicals and modulating inflammatory cytokine secretion, thereby exerting anti-inflammatory and oxidative stress-reducing effects (Hu et al., 2024). However, the precise bioactive compounds responsible for its anti-HUA effects and the associated molecular mechanisms remain unclear.

The deposition of monosodium urate (MSU) is widely recognized as a classic trigger of acute inflammatory responses and subsequent tissue injury. However, even with no MSU formation, persistently elevated soluble uric acid promotes subclinical inflammation and enhanced oxidative stress, resulting in progressive tissue damage (Yip et al., 2020). Multiple signaling pathways are involved in this process, such as AKT, NF-κB, and STAT signaling (Ge et al., 2024; Alanazi et al., 2025; Chen et al., 2026). In addition, gut-kidney axis also plays an important role in this process. Gut microbial dysbiosis is likely to disrupt short-chain fatty acid metabolism, intestinal barrier permeability, and endotoxin levels. These alternations finally lead to structural and functional renal injury (Yang et al., 2018; Tsuji et al., 2024; Deng et al., 2025). Zhang et al. reported that *Lactobacillus paracasei N1115* exerted a beneficial effect on HUA by modulating butyrate production through a cross-feeding interaction (Zhang et al., 2025). Ni et al. demonstrated that combined administration of astaxanthin and *Lactobacillus rhamnosus* could alleviate HUA via gut-kidney axis (Ni et al., 2025).

This research applied network pharmacology to explore the potential biological activities of the identified bioactive compounds. Molecular docking and molecular dynamics simulations (MDS) were utilized to construct binding models between compounds and target proteins. The findings indicated kaempferol as the primary bioactive compound contributing to the anti-HUA effect of mulberry leaf. Then a HUA mouse model was constructed to investigate the molecular mechanisms of kaempferol *in vivo*. Kaempferol exerted its therapeutic effect by reducing UA levels, modulating purine metabolism, and attenuating inflammatory responses, primarily through inhibition of AKT expression and phosphorylation. These findings offer conceptual support and novel insights into the therapeutic use of mulberry leaf for managing HUA.

## 2 Materials and methods

### 2.1 Reagents

Kaempferol (K812226) was purchased from Macklin Biochemical Co., Ltd. (Shanghai, China). Oxonic acid potassium salt (OA, 156124) and sodium carboxymethyl cellulose (CMC-Na, 419338) were obtained from Sigma-Aldrich (St. Louis, MO, USA). Allopurinol (A105386) and hypoxanthine (HX, H108384) were supplied by Aladdin Biochemical Technology Co., Ltd. (Shanghai, China). Commercial assay kits for GSH, MDA, UA, CRE, and BUN were purchased from Nanjing Jiancheng Bioengineering Institute (Nanjing, China). ELISA kits for mouse IL-1β (KT21178), IL-6 (KT99854), IL-17 (KT22800), and TNF-α (KT99985) were provided by Wuhan Moshake Biotechnology Co., Ltd. (Wuhan, China). Antibodies against AKT (#4691) and phosphorylated AKT (p-AKT, #4060) were purchased from Cell Signaling Technology (Danvers, MA, USA). Phosphatase inhibitor cocktail was purchased from Beyotime (#P1082, Beyotime, China).

### 2.2 Screening of major bioactive compounds in mulberry leaf and HUA-related targets

All bioactive compounds present in mulberry leaf were retrieved from the Traditional Chinese Medicine Systems Pharmacology Database (TCMSP, https://www.tcmsp-e.com). Compounds with oral bioavailability (OB) >30% and drug-likeness (DL) >0.18 were selected as the main bioactive compounds. The potential targets of these compounds were then predicted using the SwissTargetPrediction online platform, and those with a predicted possibility >0.1 were retained as candidate targets (Daina et al., 2019). To identify HUA-related targets, the term “hyperuricemia” was searched in the GeneCards database, yielding a total of 1,410 associated targets. Among these, 706 targets with relevance scores above the median were selected for further analysis (Stelzer et al., 2016). Additionally, 287 targets with an inference score greater than 30 were obtained from the Comparative Toxicogenomics Database (CTD, http://ctdbase.org/) (Davis et al., 2024). The overlapping targets from both databases were merged.

### 2.3 Identification of core targets of mulberry leaf against HUA

Based on the targets identified above, common targets between mulberry leaf bioactive compounds and HUA-related genes were determined. The intersecting targets were then imported into the STRING database to construct a protein-protein interaction (PPI) network, with the minimum required interaction score set to 0.4. Topological analysis of the PPI network was performed to calculate key parameters, including degree, betweenness, and closeness centralities of each node (Szklarczyk et al., 2023). Targets with all 3 topological parameters exceeding the average were identified as core targets (Supplementary Table 1).

### 2.4 Enrichment analysis

Based on the identified core targets, enrichment analyses for Gene Ontology (GO) terms and Kyoto Encyclopedia of Genes and Genomes (KEGG) pathways were conducted via the DAVID database (Sherman et al., 2022). The parameters set as “identifier = official gene symbol” and “species = Homo sapiens.” The top 10 terms in both GO and KEGG analyses were selected based on *p*-values for further interpretation.

### 2.5 Compound-target-pathway (C-T-P) network

To further explore the mechanism by which mulberry leaf exerts anti-HUA effects, the previously identified bioactive compounds, core targets, and the top 10 enriched pathways were imported into Cytoscape 3.10.3 software to construct a C-T-P network. Topological analysis of the network was conducted to calculate key metrics for each node (Supplementary Table 2).

### 2.6 Molecular docking

The 3D structures of the main compounds were retrieved from the PubChem database (Kim et al., 2023), while structures of the core target proteins were obtained from the Protein Data Bank (PDB) (Burley et al., 2023). Molecular docking between the bioactive compounds and core targets were performed using AutoDock Tools 4.0. A binding free energy of less than −7.0 kcal/mol was considered indicative of strong binding affinity (Morris et al., 2009). PyMOL software was used for visualizing ligand binding sites and interaction modes. The binding sites and docking boxes were shown in Supplementary Table 3.

### 2.7 Molecular dynamics simulations

The optimal docking complexes generated from molecular docking studies were subjected to MDS using GROMACS 2022.3 software (Van Der Spoel et al., 2005). The Amber99sb-ildn force field and tip3p water model were employed to parameterize the receptor proteins. A cubic simulation box was constructed around the receptor, and sufficient Na^+^ ions were added to neutralize the overall charge of the system. Energy minimization was performed using the steepest descent algorithm to relax all atoms in the protein structure. Equilibration was carried out in two phases: an isothermal-isochoric ensemble followed by an isothermal-isobaric ensemble, each run for 100,000 steps with a coupling constant of 0.1 picosecond (ps) and a total duration of 100 ps. Subsequently, a production MDS was conducted for 5,000,000 steps with a time step of 2 femtosecond (fs), corresponding to a total simulation time of 100 nanosecond (ns). Upon completion, the trajectory data were analyzed using built-in GROMACS tools.

### 2.8 *In vivo* experimental design

Male Kunming mice (5 weeks old, 18-22 g) were obtained from Beijing Vital River Laboratory Animal Technology Co., Ltd. (License No.: SCXK [Zhe] 2020-0002). All animals were housed in a specific-pathogen-free (SPF) environment under controlled conditions: temperature 23-25 °C, relative humidity 40-60%, and a 12 h light/dark cycle, with ad libitum access to food and water. The animal protocol was reviewed and approved by the Institutional Animal Care and Use Committee (IACUC) of Henan University of Chinese Medicine (Approval No.: IACUC-202503027). After a week of acclimatization, 36 mice were randomly divided into 6 groups (*n* = 6 per group): control group (Con), model group (Mod), allopurinol group (All, 10 mg/kg), and 3 kaempferol treatment groups: low-dose (Low, 25 mg/kg), medium-dose (Mid, 50 mg/kg), and high-dose (High, 100 mg/kg) (Qu et al., 2021). Beginning on day 8, mice in the Mod and kaempferol treatment groups were intraperitoneally injected with OA at 300 mg/kg/day (dissolved in 0.5% CMC-Na), followed 1 h later by oral administration of HX at 500 mg/kg/day, dissolved in normal saline (NS), for 14 consecutive days to induce HUA. While mice in the control group received equivalent volumes of CMC-Na or NS in the same way. Starting on day 15, all groups received their respective interventions 1 h after HX administration, once daily for 4 weeks: vehicle for the Con and Mod groups, allopurinol (10 mg/kg) for the All group, and kaempferol at different doses for the treatment groups. At the end of the treatment period, mice were anesthetized with isoflurane and euthanized by cervical dislocation. Blood samples were collected, allowed to stand at room temperature for 40 mins, and centrifuged to obtain serum, which was stored at −70 °C for subsequent analysis. The kidneys were also harvested: the left kidney was washed thoroughly and stored at −70 °C, while the right kidney was fixed in 4% paraformaldehyde for histological examination. The animal experiment design is shown in Supplementary Figure 1.

### 2.9 Biochemical index measurements

Serum levels of UA, CRE, and BUN were measured using commercial biochemical assay kits. Enzyme-linked immunosorbent assay (ELISA) kits were used to quantify the serum levels of TNF-α, IL-1β, IL-6, and IL-17. All ELISA measurements were performed in duplicate, with an intra-assay coefficient of variation (CV) < 10%. Oxidative stress in renal tissue was assessed by measuring the levels of GSH and MDA in kidney homogenates using commercial biochemical assay kits.

### 2.10 Hematoxylin and eosin (H&E) staining

Mouse right kidney tissues were adequately fixed in 4% paraformaldehyde and subsequently embedded in paraffin. Tissue blocks were sectioned into 3 μm slices, followed by H&E staining to examine histopathological changes in the kidneys across different groups.

### 2.11 Western blotting (WB)

Mouse kidney tissues were lysed on ice using RIPA buffer containing PMSF and phosphatase inhibitors to extract total protein. Proteins were separated via SDS-PAGE, transferred onto PVDF membranes, and visualized using a Bio-Rad imaging system. ImageJ software was employed to analyze band intensity, with β-actin used as the internal loading control. The expression levels of AKT and p-AKT were quantified via WB.

### 2.12 16S rRNA sequencing analysis

Total DNA was extracted from the fecal microbiota 16S rRNA gene. The V3-V4 region of 16S rRNA gene was sequenced. Amplicon Sequence Variant (ASV) accession and abundance were used for subsequent data analyses. PICRUSt2 was utilized to predict metabolic pathways and cluster of orthologous group (COG) functional categories of the microbiota.

### 2.13 Correlation analysis

To identify potential key microbial taxa, we performed correlation analyses between dominant differential microbes and the altered key indicators. Pearson correlation coefficients were applied to assess the value of association between microbiota taxa and clinical variables.

### 2.14 Statistical analysis

All experimental data were expressed as mean ± standard deviation (mean ± SD). Statistical analysis was performed using SPSS 26.0 software (IBM, Chicago, USA). One-way analysis of variance was applied for intergroup comparisons, followed by Tukey's *post hoc* test. A *p*-value of less than 0.05 was considered statistically significant.

## 3 Results

### 3.1 Screening of bioactive compounds in mulberry leaf and their associated targets against HUA

**Figure 1:**
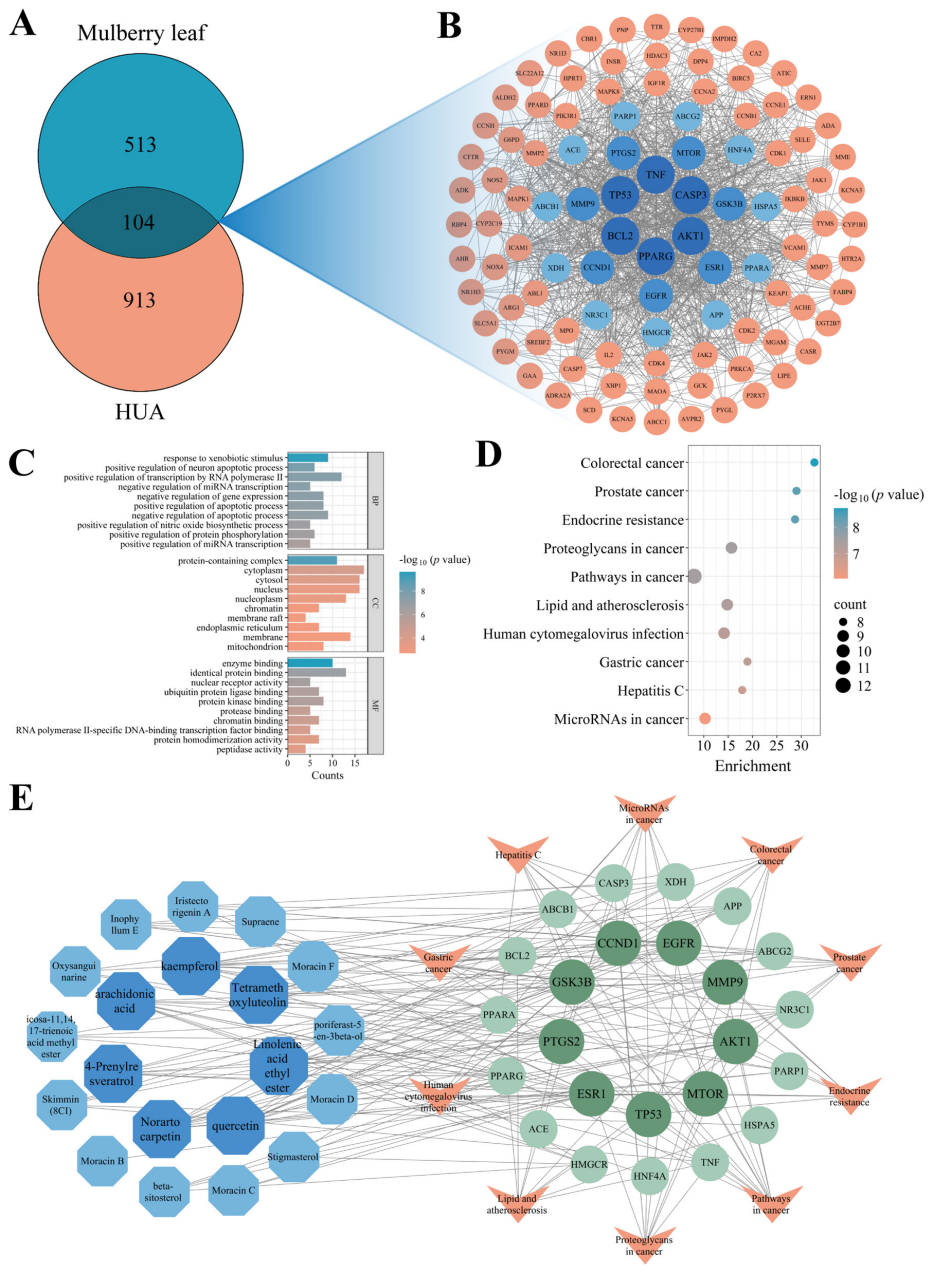
Network pharmacology analysis of mulberry leaf against HUA. **(A)** Overlapping targets between mulberry leaf and HUA; **(B)** PPI network of the overlapping targets; **(C)** GO enrichment and **(D)** KEGG pathway analysis of the 24 core targets; **(E)** C-T-P network diagram (blue polygons: bioactive compounds; green circles: core targets; orange triangles: signaling pathways).

A total of 269 chemical compounds of mulberry leaf were retrieved from the TCMSP database. The SMILES structures of these compounds were submitted to the SwissTargetPrediction web server to predict their potential targets. The resulting targets were then cross-referenced with the UniProt database to standardize protein names. A total of 513 unique targets related to the bioactive compounds were identified. Meanwhile, HUA-related disease targets were obtained, resulting in 913 non-redundant targets. A Venn diagram analysis revealed 104 overlapping targets between the compound-related targets and HUA-related disease targets, indicating potential anti-HUA relevance (Figure 1A).

### 3.2 Construction of the PPI network

A PPI network was constructed based on the 104 intersecting targets, and after removing the disconnected nodes, the resulting PPI network was constructed, consisting of 102 nodes and 1,176 edges (Figure 1B). The average degree, betweenness, and closeness centrality values of the intersecting targets were 23.06, 94.76, and 0.53, respectively, and 24 targets were identified as core targets. Among them, AKT1, TNF, TP53, BCL2, CASP3, and PPARG degree value exceeded 60, suggesting that these targets may serve as more critical molecular targets of mulberry leaf in the treatment of HUA.

### 3.3 Enrichment analysis of core targets

To further investigate the biological functions of the core targets, GO and KEGG analyses were conducted using the DAVID database (Figures 1C, D). GO enrichment results revealed that the core targets were involved in a wide range of BP, CC, and MF. Using a significance threshold of *p* < 0.05, 173 BP terms were identified. The top 3 was: response to xenobiotic stimulus, positive regulation of neuron apoptotic process, and positive regulation of transcription by RNA polymerase II. A total of 22 CC terms were enriched, with the top 3 being protein-containing complex, cytoplasm, and cytosol. For MF, 65 terms were identified, mainly associated with enzyme binding, identical protein binding, and nuclear receptor activity. These findings suggest that the anti-HUA effects of mulberry leaf may be closely related to the regulation of enzyme activity and resistance to oxidative stress. KEGG pathway analysis revealed that 81 signaling pathways were significantly enriched. The top 10 enriched pathways were visualized and primarily involved: colorectal cancer, prostate cancer, endocrine resistance, proteoglycans in cancer, pathways in cancer, lipid and atherosclerosis, human cytomegalovirus infection, gastric cancer, hepatitis C, and microRNAs in cancer.

### 3.4 C-T-P network analysis

Using Cytoscape software, a network was constructed, consisting of 25 bioactive compounds from mulberry leaf, 24 core targets, and the top 10 enriched signaling pathways. A total of 5 compounds that were not connected to any core targets were excluded, resulting in a finalized C-T-P network (Figure 1E). The resulting network contained 54 nodes and 194 edges. Topological parameter analysis revealed an average degree of 7.19, average betweenness centrality of 80.78, and average closeness centrality of 0.40. Based on degree ranking, the 5 most important bioactive compounds were identified as kaempferol, norartocarpetin, arachidonic acid, 4-prenylresveratrol, and quercetin.

### 3.5 Molecular docking analysis

**Figure 2:**
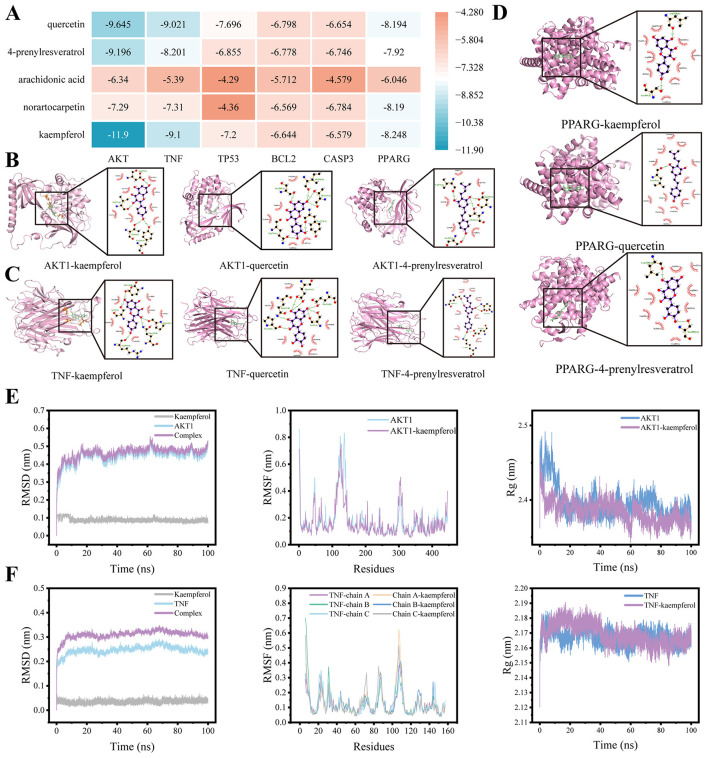
Molecular docking and MDS analysis. **(A)** Binding energies of the 3 bioactive compounds with the core targets; **(B)** AKT1, **(C)** TNF and **(D)** PPARG form hydrogen bonds with residues of core targets, respectively. **(E)** RMSD, RMSF, and Rg analysis of AKT1-kaempferol and **(F)** TNF-kaempferol.

To validate the interactions between the key bioactive compounds and core targets, molecular docking was performed to assess binding affinities. The top 5 bioactive compounds were selected as ligands, while 6 most critical molecular targets served as receptor proteins. As shown in Figure 2A, among all docking models, AKT-kaempferol and TNF-kaempferol demonstrated the strongest binding affinities. Visualization of target-compound interactions and binding modes was performed using PyMOL (Figures 2B-D). The results revealed that kaempferol formed hydrogen bonds with key amino acid residues of AKT (VAL-271, ASP-292, and TYR-326) and TNF (LYS-98, GLN-102, and GLU-116). These findings confirm that the bioactive compounds in mulberry leaf exhibit strong binding potential with critical HUA-related targets.

### 3.6 Molecular dynamics simulations analysis

To further evaluate the stability of interactions between kaempferol and the two key targets, AKT and TNF, 100 ns MDS were conducted for the 2 best docking complexes: AKT-kaempferol and TNF-kaempferol (Figures 2E, F). The RMSD curves showed the overall stability and conformational fluctuations of both complexes. In comparison to the protein-solvent systems alone, all systems exhibited initial fluctuations during the first 20 ns, after which they stabilized, indicating the systems reached equilibrium under the simulation conditions. The total complexes (black curves) showed greater fluctuations than the corresponding apo-proteins (red curves), likely due to conformational changes induced by ligand binding. This suggests that kaempferol binding affects the structural stability of AKT and TNF, although both systems eventually achieved a relatively stable conformation after 20 ns. The RMSF plots reflected the flexibility of amino acid residues within the complexes. Peaks near residues 120 and 310 indicated regions of higher flexibility, possibly corresponding to loops or substrate-binding regions near the active site. Increased flexibility in these areas may enhance ligand interaction and facilitate functional adaptability of the protein. In contrast, lower RMSF values in other regions suggest that much of the complex remains structurally stable, preserving the enzyme's core conformation. The Rg plots were used to assess the compactness of the AKT1-kaempferol and TNF-kaempferol complexes. The consistently stable Rg values observed during the simulation suggested that the tertiary structures of the complexes remained largely unchanged, with no marked expansion or contraction, thereby reinforcing the structural stability of the target-compound complexes.

### 3.7 Kaempferol alleviates HUA in mice

**Figure 3:**
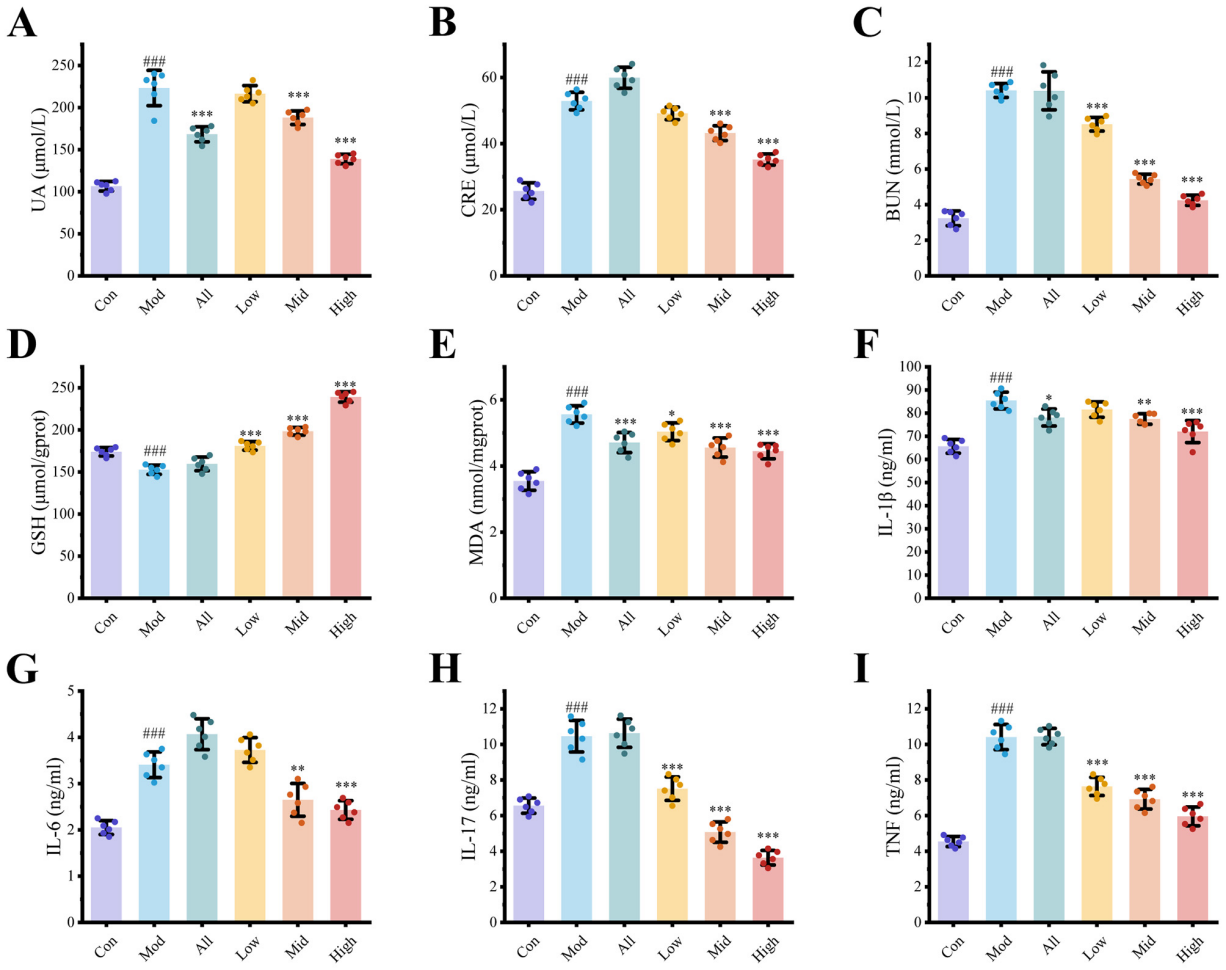
Effects of kaempferol against HUA. **(A)** Serum UA levels after treatment; **(B, C)** Serum renal function indicators: CRE and BUN; **(D, E)** Oxidative stress markers in kidney tissue: GSH and MDA; **(F-I)** Levels of inflammatory cytokines in serum. Each dot represents the measurement from an individual mouse (*n* = 6 per group), and bars indicate the mean ± SD. Statistical significance was determined by one-way ANOVA followed by Tukey's *post hoc* test. ^*^p < 0.05, ^**^p < 0.01, ^***^p < 0.001 vs. Mod group; ^*###*^p < 0.001 vs. Con group.

HUA mouse model was employed to explore the mechanism of kaempferol in managing HUA. In the High group, serum UA levels were reduced by 37.81% (*p* < 0.001), indicating that kaempferol exerts potent anti-HUA activity in mice (Figure 3A). Additionally, CRE and BUN levels were significantly reduced by 33.49% and 59.25%, respectively, in the High group (*p* < 0.001), suggesting a potential reno-protective effect of kaempferol (Figures 3B, C). Subsequently, oxidative stress markers and inflammatory cytokines were assessed in kidney tissues. High dose kaempferol treatment significantly increased the level of the antioxidant GSH by 56.62% (*p* < 0.001) and reduced the level of MDA by 20.05% (*p* < 0.001), indicating enhanced renal antioxidant capacity and reduced oxidative stress (Figures 3D, E). As for inflammatory cytokines, the most notable was IL-17 (Figure 3H), which decreased by 51.50% in the Mid group and 65.25% in the High group (*p* < 0.001). Additionally, other cytokines also showed varying degrees of reduction: IL-1β (Figure 3F) decreased by 15.68%, IL-6 (Figure 3G) by 28.72%, and TNF-α (Figure 3I) by 42.79% (*p* < 0.001).

### 3.8 Histopathological effects of kaempferol on kidney tissue in mice

**Figure 4:**
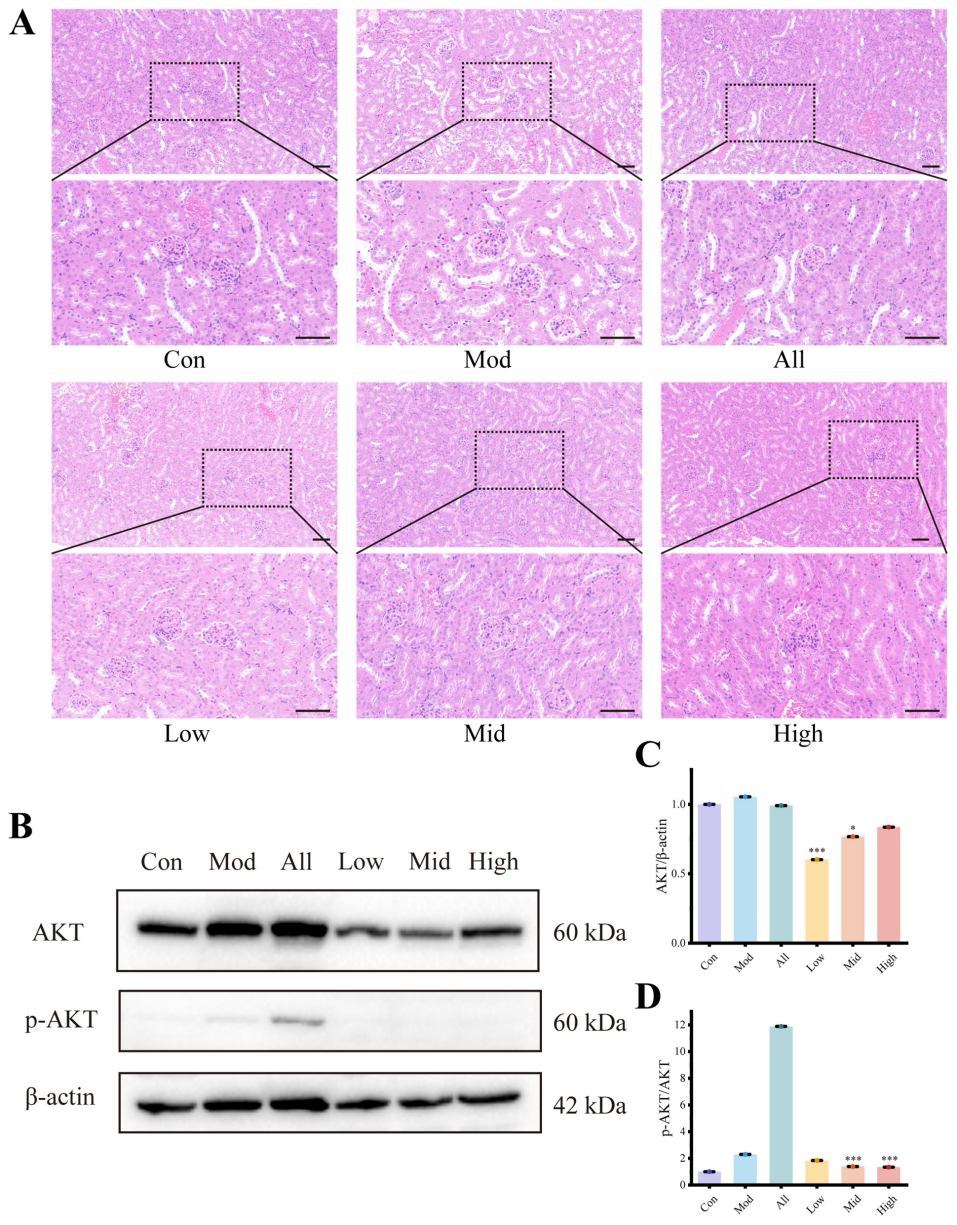
Kaempferol ameliorates renal injury and inhibits the AKT Pathway. **(A)** Representative H&E-stained kidney sections from each group after treatment (bar = 50 μm); **(B)** WB images of total AKT and p-AKT; **(C, D)** quantification of the relative expression levels of total AKT and p-AKT based on grayscale analysis of the blotting results. ^*^p < 0.05, ^***^p < 0.001 vs. Mod group.

As shown in Figure 4A, H&E staining revealed significant renal damage in HUA model mice induced by combined administration of OA and HX. Pathological features included varying degrees of tubular epithelial cell swelling and degeneration, mild tubular dilation, mesangial cell proliferation, and inflammatory cell infiltration. Notably, these pathological alterations were markedly alleviated in the high-dose kaempferol treatment group, suggesting a protective effect of kaempferol against UA-induced renal injury.

### 3.9 Effects of kaempferol on core targets

Based on the above results, AKT was identified as the major potential target through which kaempferol exerts its therapeutic effects. To validate this, the expression levels of AKT and p-AKT in renal tissue were evaluated by WB (Figure 4B). The results revealed that total AKT protein levels remained unchanged in model mice, whereas p-AKT expression was significantly increased, and the p-AKT/AKT ratio increased by 129.47% compared with the control group. Then, in kaempferol treated mice, decreases were observed in both AKT and p-AKT expression. And a 41.51% decrease of p-AKT/AKT ratio was observed in the High group compared with the model group, indicating that kaempferol effectively inhibits AKT expression and phosphorylation in HUA mice (Figures 4C, D).

### 3.10 Kaempferol attenuated gut microbiota of HUA mice

**Figure 5:**
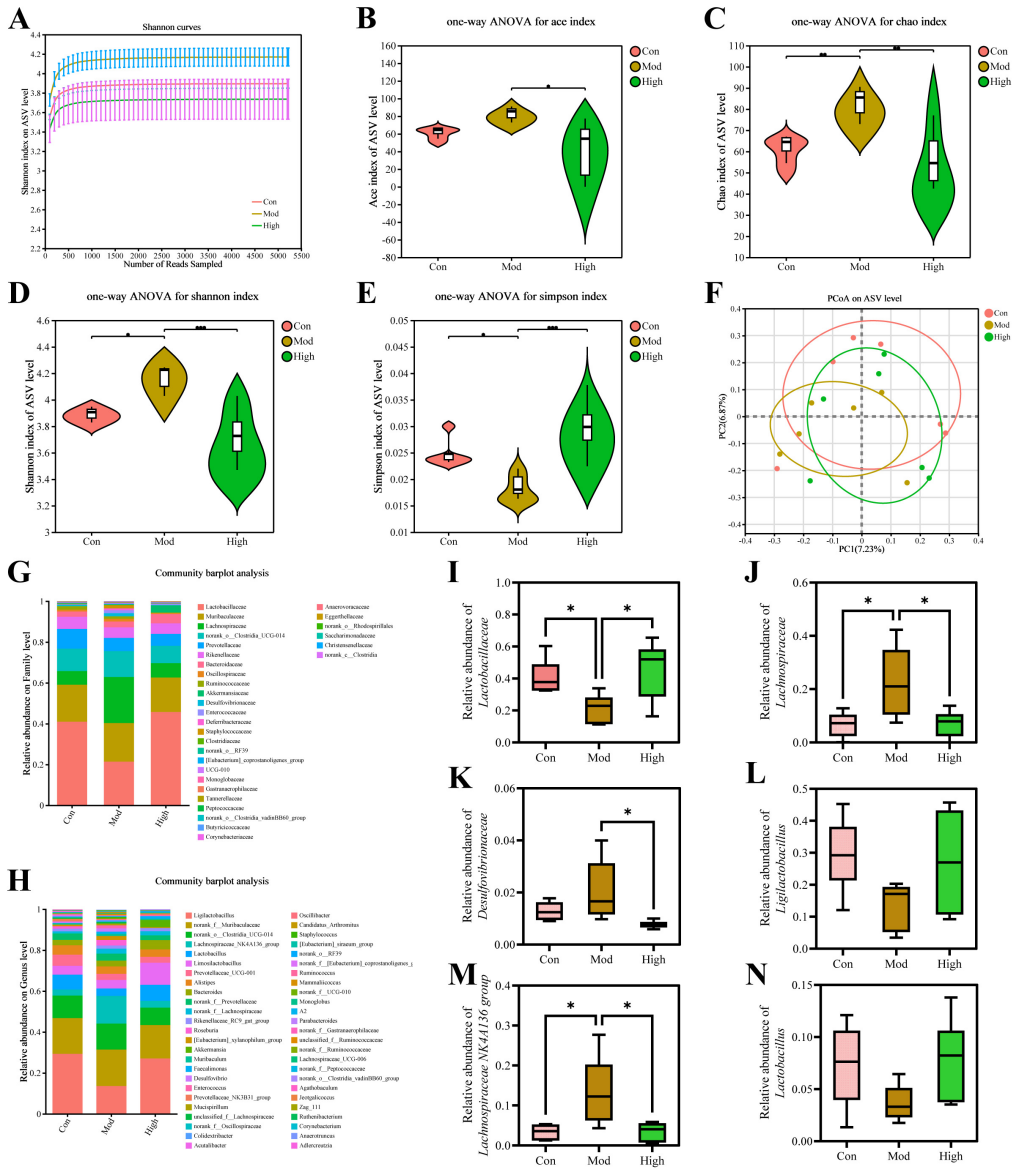
Kaempferol modulate gut microbiota diversity and composition on ASV level. **(A)** Shannon rarefaction curves. **(B-E)** Violin plots of ACE index, Chao1 index, Shannon index, and Simpson index. Statistical significance was determined by one-way ANOVA**. (F)** PCoA β-diversity analysis. **(G, H)** Community barplot analysis at family and genus levels. **(I-K)** Relative abundance of representative microbiomes at family level. **(L-N)** Relative abundance of representative microbiomes at genus level. Data are presented as mean ± SD. *p* < 0.05 indicates statistically significant differences between groups. ^*^p < 0.05.

As the high-dose group showed superior outcomes in biochemical indices and histopathological analysis compared with the other treatment groups, it was chosen for subsequent investigations. To characterize the effects of kaempferol on the gut microbiota of HUA mice, fecal samples were prepared to 16S rRNA sequencing following the aforementioned procedure. The Shannon index gradually increased and then reached a plateau, indicating the results had sufficient representativeness (Figure 5A).

As shown in Figures 5B-E, the α-diversity index in Mod group significantly changed, and kaempferol treatment modulated these indices toward Con group profile. PCoA analysis revealed that the gut microbiota structure in the Mod group changes compared with the Con group, while it shifted toward Con group after treatment with kaempferol, suggesting that kaempferol exerted a restorative effect on gut microbial composition (Figure 5F).

At the family level, kaempferol treatment reversed the abundance alterations of *Lactobacillaceae, Lachnospiraceae* and *Desulfovibrionaceae* caused by HUA (Figures 5G, I-K). In this study, both of *Lachnospiraceae* and *Desulfovibrionaceae* showed a significant increase in the Mod group, which was alleviated by kaempferol treatment. Similarly, at the genus level, kaempferol also showed a clear regulatory trend on the abundances of several genus (Figures 5H, L-N). HUA induced microbiota dysbiosis in mice, and kaempferol altered these changes at family and genus levels.

### 3.11 Correlation analysis and functional prediction of differential microbiota

**Figure 6:**
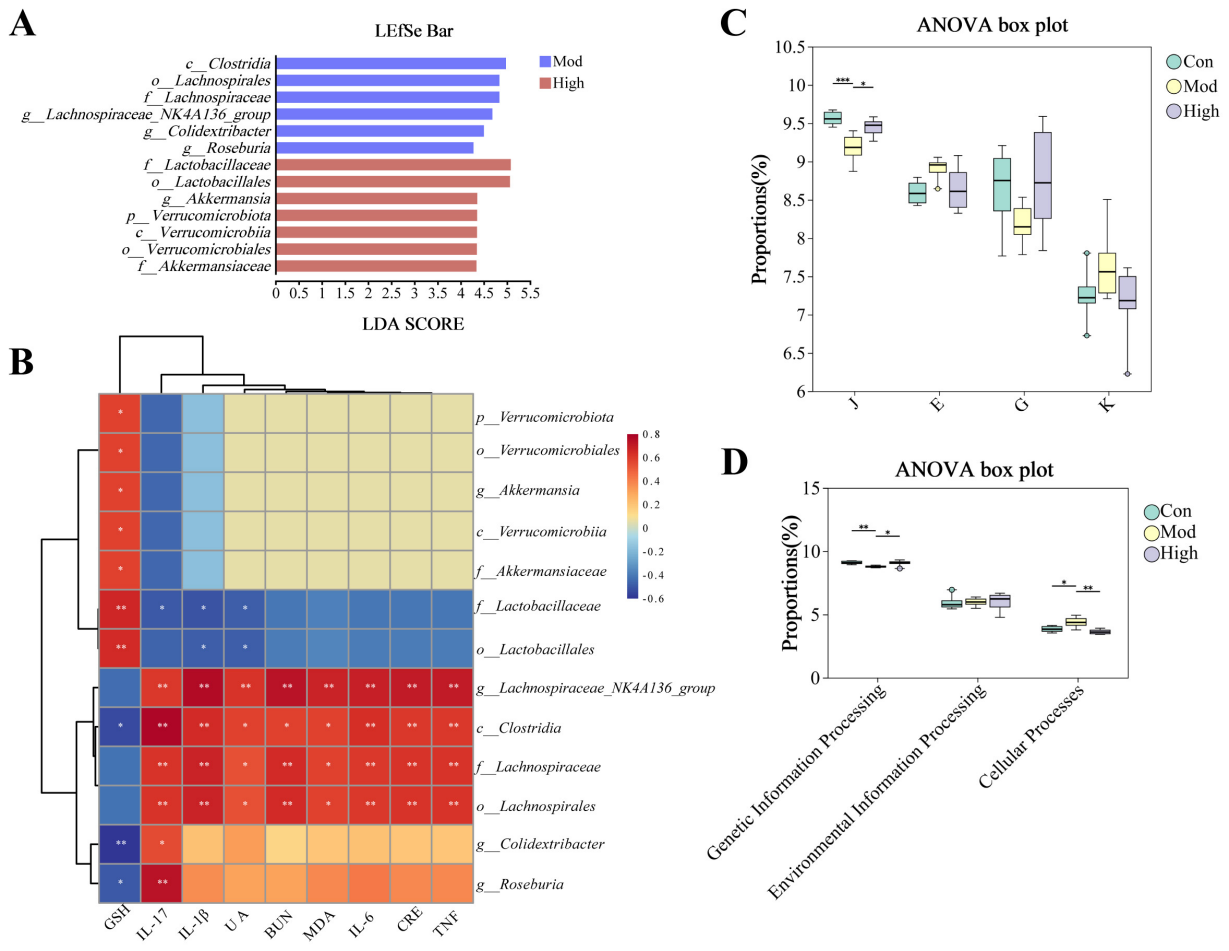
Differential analysis and functional correlation of gut microbiota. **(A)** LEfSe analysis (LDA > 2). **(B)** Spearman correlation heatmap between significantly altered microbiomes and biochemical indicators (GSH, IL-17, IL-1β, UA, BUN, MDA, IL-6, CRE, and TNF). **(C)** COG functional categories analysis. **(D)** KEGG functional pathway analysis. ^*^p < 0.05, ^**^p < 0.01, ^***^p < 0.001.

To explore the relationship between gut microbiota alterations and the progression of HUA, we performed a correlation analysis between microbiota composition and HUA-related cytokines. LEfSe analysis was used to identify the differences in gut microbiota among the fecal samples of mice from different groups (Figure 6A). In the Mod group, *Lachnospiraceae_NK4A136_group, Colidextribacter*, and *Roseburia* were identified as the dominant genus. In contrast, after kaempferol treatment, *Lactobacillaceae* and *Akkermansiaceae* exhibited higher relative abundances compared to the Mod group. Pearson's correlation analysis revealed that *Lachnospiraceae_NK4A136_group, Colidextribacter*, and *Roseburia* were positively correlated with the progression of HUA, whereas *Lactobacillaceae* and *Akkermansiaceae* showed a negative correlation (Figure 6B). UA and IL-1β were negatively correlated with *Lactobacillaceae*, but positively correlated with *Lachnospiraceae_NK4A136_group*. IL-17 showed a significant negative correlation with *Lactobacillaceae*, while exhibiting significant positive correlations with *Lachnospiraceae_NK4A136_group, Colidextribacter*, and *Roseburia*. GSH levels were significantly negatively correlated with *Colidextribacter* and *Roseburia*, but positively correlated with *Lactobacillaceae* and *Akkermansiaceae*. Moreover, *Lachnospiraceae_NK4A136_group* displayed significant positive correlations with several cytokines, including CRE, BUN, IL-6, TNF-α, and MDA.

Subsequently, functional prediction was performed using PICRUSt2. Based on the COG classification system, differences in microbial functions were analyzed (Figure 6C). The Mod group showed a significant decrease in the J category, compared with the Con group. Kaempferol treatment significantly attenuated the alteration. KEGG pathway enrichment indicated the microbiota in Mod group significantly altered in genetic information processing and cellular processes, and kaempferol treatment counteract these alterations (Figure 6D).

## 4 Discussions

Elevated serum UA levels, along with increased oxidative stress and dysregulated inflammatory cytokines, are key pathological features of HUA. Mulberry leaf, rich in flavonoids and polyphenolic compounds, has potent antioxidant, anti-inflammatory, and metabolic regulatory properties, including modulation of blood glucose and lipid levels (Shan et al., 2022; Shi et al., 2022). In addition, mulberry leaf has been shown to lower serum UA in HUA model mice, potentially through inhibition of xanthine oxidase activity, suggesting great therapeutic potential in the prevention and treatment of HUA (Wang et al., 2025). This study integrated network pharmacology, molecular docking, and MDS to investigate the major anti-HUA compounds of mulberry leaf and the potential mechanisms. Kaempferol is probably a principal active compound which is responsible for the anti-HUA effects of mulberry leaf. AKT1 and TNF were identified as its core molecular targets.

Kaempferol is a naturally occurring flavonoid widely distributed in various plants, fruits, and vegetables. It is distinguished by its antitumor, antioxidant, and anti-inflammatory properties (Šelo et al., 2022; Zhang et al., 2022b). Numerous studies have demonstrated that kaempferol exhibits safety at biologically relevant doses, which make it a promising candidate as a natural therapeutic agent (Mamouni et al., 2018; Crocetto et al., 2021). Recent findings have shown that kaempferol can effectively ameliorate HUA by enhancing UA excretion through the regulation of key urate transporters, including ABCG2, OCT2, OAT1, and GLUT9 (Huang et al., 2024). Also, kaempferol derivatives, such as kaempferol3′-sulfonate and kaempferol-34′-di-O-β-glucoside, have been demonstrated *in vitro* inhibitory activity against xanthine oxidase (Teixeira et al., 2022; Wang et al., 2023a). These findings further underscore the therapeutic potential of kaempferol and its derivatives in the treatment of HUA.

PI3K/AKT signaling pathway is critically involved in the pathogenesis and advancement of HUA (Wan et al., 2025). AKT is known to catalyze the phosphorylation of URAT1 at the Thr408 site, a modification that promotes glycosylation and enhances the membrane localization of URAT1. This, in turn, increases URAT1 activity and facilitates renal reabsorption of UA (Fujii et al., 2025). Moreover, NF-κB is closely linked to AKT signaling (Grandage et al., 2005; Yang et al., 2019a; Chang et al., 2021). Earlier research has demonstrated that isorhamnetin can inhibit the PI3K/AKT/NF-κB pathway and thereby alleviate UA-induced renal inflammation in mice (Kong et al., 2025). Similarly, corn silk flavonoids have been reported to attenuate HUA by downregulating this pathway, thereby reducing inflammation and apoptosis (Wang et al., 2023b). Taken together, the inhibition of AKT signaling can modulate renal UA transport, oxidative stress, and inflammation, ultimately contributing to HUA mitigation. TNF-α is a key proinflammatory cytokine that activates the MAPK and NF-κB pathways. It can also initiate apoptosis via the TNFR1 death domain, a mechanism implicated in tissue damage during HUA progression (Qin et al., 2012). In this study, the phosphorylation of AKT increased in the kidneys of HUA mice. Kaempferol treatment significantly suppressed the expression and phosphorylation of AKT proteins, corroborating the computational predictions and supporting its role in targeting these critical pathways in the context of HUA. However, due to the low initial expression of TNF, we did not detect sufficient expression of TNF in mouse kidney tissues by WB experiment. Instead, a significant decrease in TNF-α after kaempferol administration were observed in ELISA assay.

As one of the most common probiotic families, *Lactobacillaceae* protect the intestinal barrier and modulate inflammatory responses (Walter and O'Toole, 2023). Kaempferol treatment effectively alleviated the dysbiosis of *Lactobacillaceae* observed in the Mod group. Similarly, another up-regulated family is *Lachnospiraceae*. Certain strains of *Lachnospiraceae* exhibit potential toxicity and may be associated with chronic inflammation (Vacca et al., 2020). Previous studies have reported a significant increase in *Lachnospiraceae* abundance in chronic kidney disease (CKD) (Ren et al., 2023). In this research, the accumulation of kaempferol reversed the alternation in the composition of *Lachnospiraceae*. The excessive proliferation of *Desulfovibrionaceae* indicates the occurrence of inflammatory responses and the disruption of intestinal homeostasis (Jin et al., 2025). Previous studies have shown that *Akkermansiaceae* exhibited a decreased abundance in HUA mouse and was negatively correlated with intestinal permeability (Lv et al., 2023). Similarly, Yin et al. also reported an elevated abundance of *Akkermansiaceae* during the treatment of hyperuricemia with fermented *Artemisia selengensis* Turcz extracts. In this study, *Akkermansiaceae* showed a positive correlation with GSH, suggesting that this bacterial family may help restore oxidative stress homeostasis in the host.

In conclusion, this study indicated kaempferol might be an important compound for mulberry leaf in managing HUA. Mechanistically, kaempferol reduced AKT expression and phosphorylation and lowered TNF-α expression, which is potentially linked to the attenuation of oxidative stress and inflammatory responses in renal tissues. Moreover, kaempferol remodeled gut microbiota, and restored microbiota homeostasis.

## Data Availability

All data of this article are included within the article. It can also be requested from the corresponding author or first author. The sequencing data have been deposited in the NCBI Sequence Read Archive (SRA) under accession number PRJNA1393186 (BioProject). The dataset includes 18 BioSamples and their corresponding SRA accessions, which can be accessed at https://www.ncbi.nlm.nih.gov/bioproject/PRJNA1393186.
