# Supplementary material for: Unraveling the mechanism of mulberry leaf in alleviating hyperuricemia: key role of kaempferol by modulating AKT pathway and gut-kidney axis
Source: Front Microbiol. 2026 Jan 22;17:1752775. doi: 10.3389/fmicb.2026.1752775 (PMC12872837; doi:10.3389/fmicb.2026.1752775)
Supplement: Supplementary file 1 [file Table_1.docx]

**Supplemental table 1 The topological parameters of 24 core targets**

| Targets | Betweenness | Closeness | Degree |
| --- | --- | --- | --- |
| AKT1 | 838.1565 | 0.765152 | 71 |
| TNF | 893.2602 | 0.753731 | 69 |
| TP53 | 460.2758 | 0.731884 | 66 |
| BCL2 | 652.0852 | 0.711268 | 61 |
| CASP3 | 236.9085 | 0.701389 | 60 |
| PPARG | 475.7216 | 0.706294 | 60 |
| ESR1 | 445.6008 | 0.682432 | 56 |
| EGFR | 372.0661 | 0.673333 | 54 |
| MMP9 | 256.0957 | 0.664474 | 53 |
| PTGS2 | 321.4862 | 0.660131 | 51 |
| CCND1 | 109.3277 | 0.63125 | 47 |
| GSK3B | 170.4434 | 0.63522 | 46 |
| MTOR | 101.117 | 0.63125 | 46 |
| PARP1 | 120.687 | 0.608434 | 42 |
| PPARA | 158.9237 | 0.615854 | 42 |
| ACE | 389.2838 | 0.60119 | 36 |
| HSPA5 | 122.5788 | 0.583815 | 34 |
| APP | 135.1717 | 0.577143 | 33 |
| NR3C1 | 168.3882 | 0.577143 | 31 |
| HMGCR | 105.3346 | 0.58046 | 30 |
| XDH | 217.3831 | 0.58046 | 29 |
| ABCB1 | 171.7141 | 0.570621 | 28 |
| HNF4A | 102.1126 | 0.551913 | 26 |
| ABCG2 | 231.6699 | 0.561111 | 26 |

**Supplemental table 2 Topological analysis of the 5 compounds in C-T-P network**

| Compounds | Betweenness | Closeness | Degree |
| --- | --- | --- | --- |
| kaempferol | 161.1457692 | 0.473214286 | 11 |
| Norartocarpetin | 161.1457692 | 0.473214286 | 11 |
| arachidonic acid | 342.128371 | 0.481818182 | 10 |
| quercetin | 73.87412511 | 0.4140625 | 9 |
| 4-Prenylresveratrol | 112.2368877 | 0.456896552 | 8 |

**Supplemental table 3 Topological analysis of the 5 compounds in C-T-P network**

| Docking models | Center (x, y, z) | Docking size (x, y, z) |
| --- | --- | --- |
| AKT-kaempferol | 5, -7, 13 | 26, 26, 26 |
| AKT-norartocarpetin | 5, -7, 13 | 26, 26, 26 |
| AKT-arachidonic acid | 5, -7, 13 | 26, 26, 26 |
| TNF-kaempferol | 8, 63, 31 | 26, 26, 26 |
| TNF-norartocarpetin | 8, 63, 31 | 26, 26, 26 |
| TNF-arachidonic acid | 8, 63, 31 | 26, 26, 26 |
| TP53-kaempferol | 78, 28, 46 | 26, 26, 26 |
| TP53-norartocarpetin | 78, 28, 46 | 26, 26, 26 |
| TP53-arachidonic acid | 78, 28, 46 | 26, 26, 26 |
