# Supplementary figures and images for: Unraveling the mechanism of mulberry leaf in alleviating hyperuricemia: key role of kaempferol by modulating AKT pathway and gut-kidney axis
Source: Front Microbiol. 2026 Jan 22;17:1752775. doi: 10.3389/fmicb.2026.1752775 (PMC12872837; doi:10.3389/fmicb.2026.1752775)

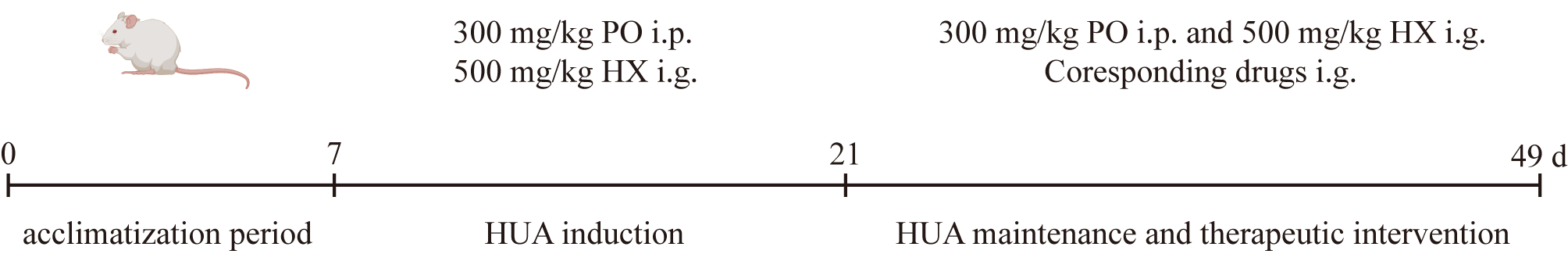

Supplement: Supplementary file 2 [file Image_1.tif]
